# Draft genome sequence of *Wigglesworthia glossinidia* “palpalis gambiensis” isolate

**DOI:** 10.1128/mra.00912-23

**Published:** 2024-01-11

**Authors:** Noah Spencer, Mathilda Santee, Adam Wetherhold, Rita V. M. Rio

**Affiliations:** 1 Department of Biology, Eberly College of Arts and Sciences, West Virginia University, Morgantown, West Virginia, USA; 2 Biodesign Center for Mechanisms of Evolution and School of Life Sciences, Arizona State University, Tempe, Arizona, USA; Indiana University, Bloomington, USA

**Keywords:** tsetse fly, obligate mutualist, *Wigglesworthia*, ancient endosymbiont

## Abstract

The 0.719 Mb genome of the tsetse endosymbiont, *Wigglesworthia glossinidia,* from *Glossina palpalis gambiensis* is presented. This *Wigglesworthia* genome retains 611 protein-coding sequences and a 25.3% GC content. A cryptic plasmid is conserved, between *Wigglesworthia* isolates, suggesting functional significance. This genome adds a further dimension to characterize *Wigglesworthia* lineage-based differences.

## ANNOUNCEMENT

Tsetse harbor an obligate endosymbiont, *Wigglesworthia glossinidia* (1), within bacteriomes involved in nutritional supplementation (2–4) and immunological priming (5–7). Here, we present the *Wigglesworthia glossinidia* genome isolated from riverine *Glossina palpalis gambiensis*.

*G. palpalis gambiensis* pupae were reared to adults. Five bacteriomes per library were dissected, pooled, and total DNA isolated using the Epicentre Masterpure DNA Purification Kit. DNA sequencing was performed at the Microbial Genome Sequencing Center (now SeqCenter) in Pittsburgh, PA, USA.

Libraries were prepared using an Illumina Nextera Kit and sequenced on an Illumina NextSeq 550. The 2 × 150 bp paired-end reads were processed using Trim Galore v. 0.6.1 (8) (parameters-*q10 –length 20*) to remove adapters and low-quality base calls (Phred score <10). Pairs of trimmed reads were assembled using SPAdes v.3.15.3 (– *meta* mode) (9). To remove contamination from tsetse and its facultative symbiont *Sodalis glossinidius,* contigs were filtered using SprayNPray v. 1.0 (10) to those for which at least 50% of predicted protein sequences had a top DIAMOND (v. 2.0.4.142) hit (11) to *Wigglesworthia* in the RefSeq non-redundant protein database. Reads were then mapped back to these putatively *Wigglesworthia-*derived contigs using the Burrows-Wheeler Alignment-MEM algorithm 0.7.17 (parameters -*k 50 -B 15 -O 15 T 150*) (https://arxiv.org/abs/1303.3997). FASTQ files corresponding to the mapped reads (56.6% of total trimmed reads) were used for final SPAdes assembly (–*careful* mode), and output contigs with length >2,000 bp and mean coverage >1,400× were oriented based on the *Wigglesworthia “*morsitans” genome (NC_016893.1) using the CSAR-web server (12). Putative protein-coding sequences (CDS) and non-coding RNAs were identified with Prodigal v2.6.3 and Rfam v14.7, respectively (13–15). Overlapping sequences and gene features in the ordered contigs were used to produce the final scaffolds. Default parameters were used unless specified.

The final genome assembly totals 14 contigs with an N50 of 74 kb. The *Wigglesworthia glossinidia “*palpalis gambiensis” genome size (chromosome >0.713 Mb) and GC content (25.3%) are similar to *Wigglesworthia* described in *Glossina brevipalpis* (0.698 Mb, 22% G + C) and *G. morsitans* (0.719 Mb, 22.5% G + C) (16, 17) (Table 1). A total of 611 putative protein-coding genes, 6 rRNA genes, and 34 tRNA genes were identified. Pseudofinder v1.1.0 (18) identified 13 candidate pseudogenes with 6 in metabolic pathways as identified through KEGG Mapper (19). One gene, tRNA (M7G46)-methyltransferase *trmB,* is found in the genome but missing in other sequenced *Wigglesworthia*. A cryptic 5,326-bp plasmid containing six syntenic ORFs is retained by all *Wigglesworthia*. Using DNA alignments of the plasmid ORFs from all three *Wigglesworthia* genomes, we found rates of non-synonymous and synonymous substitutions were inconsistent with relaxed selection (dN/dS <0.2) (20). This, combined with the active transcription of plasmid genes (21), suggests that the plasmid is important to the symbiosis.

**TABLE 1:** Genome features of the *Wigglesworthia* isolates

	Wgb16^*a*^	Wgm17^*b*^	Wgp^*c*^
Chromosome, Mb	0.698	0.719	0.719
Plasmid, bp	5,280	5,198	5,326
G + C ratio, %	22	22.5	25.3
Protein coding, %	89	83.9	82.1
Predicted CDS	618	620	611
rRNA operons	2	2	2
tRNAs	34	34	34
Average ORF length, bp	988	979	975
Pseudogenes	11	8	13

^*a*^ Wgb: *Wigglesworthia* isolated from *Glossina brevipalpis*, accession numbers: BA000021.3 (chromosome), AB063523.1 (plasmid).

^*b*^ Wgm: *Wigglesworthia* isolated from *Glossina morsitans*, accession numbers: CP003315.1 (chromosome), N/A (plasmid).

^*c*^ Wgp: *Wigglesworthia* isolated from *Glossina palpalis gambiensis,* accession numbers: JAVBJL010000010.1 (chromosome and plasmid).

To date, *Wigglesworthia* genome sequences of the forest *G. brevipalpis* and savannah *G. morsitans* are available (16, 17). Although high gene conservation and synteny occur, small metabolic differences impact tsetse vector competency (22). The availability of the *Wigglesworthia glossinidia* “palpalis gambiensis” genome adds a further dimension to characterize lineage-based differences.

## Data Availability

The draft genome sequence of *Wigglesworthia* palpalis gambiensis (accession no. JAVBJL000000000.1) and raw sequencing reads (accession nos. SRR21669497 and SRR21669498) have been deposited in NCBI Genbank under BioProject PRJNA882997. Reads from both SSR entries were incorporated in the final assembly to enhance coverage.
